# Rethink International Intervention. Afghanistan, Mali, and Now?

**DOI:** 10.1007/s12399-022-00888-7

**Published:** 2022-04-11

**Authors:** Ursula Werther-Pietsch

**Affiliations:** Institut für Völkerrecht und internationale Beziehungen, University of Graz, Universitätsstraße 15A, 8010 Graz, Austria

**Keywords:** Peace and security architecture, International crisis and conflict management, Geopolitics, Transition, Autocratisation, Whole-of-government approach, Sicherheitsarchitektur, Internationales Krisen- und Konfliktmanagement, Geopolitik, Transition, Autokratisierung, Gesamtstaatlicher Ansatz

## Abstract

The recent take-over of the Taliban regime in Afghanistan marks a turn in international crisis and conflict management. The configuration as a double-hatted mission to secure and rebuild on the one hand, and in countering insurgency and terrorism on the other finally failed, an outcome that seems to recur in Mali. International law of transition and whole-of-system approaches remain however hypercritical work on the ground. A successful flexible framework for sustainable conflict transformation and peace processes must absorb lessons of both autocratic *realpolitik* and a massive trend of bipolarisation of geopolitics.

## Abstract (de)

Die jüngste Übernahme des Talibanregimes in Afghanistan zeichnet einen Wendepunkt im internationalen Krisen- und Konfliktmanagement. Der Doppelansatz aus Stabilisierung und Wiederaufbau auf der einen Seite und Terrorismusbekämpfung auf der anderen ist letztlich gescheitert – ein Vorgang, der sich in Mali zu wiederholen scheint. Ein internationales Transitionsrecht braucht, um dem „gesamtstaatlichen“ Ansatz einen Handlungsrahmen vorzugeben und ihn damit erst wirksam zu machen, ein flexibles Regelwerk für Konfliktbearbeitung und Friedensprozesse. Dieses sollte die Lektionen sowohl der Realpolitik und autokratischer Trends beachten, als auch einer massiven geopolitischen Bipolarisierung beachten.

## Future of democracies – introduction

According to the CIVICUS Monitor (2021), 87% of the world’s population now lives in countries in which the governance and public space are classified as “closed”, “repressed” or “obstructed” – an increase of over 4% since 2020. Leading think-tanks like V‑Dem and Freedom House made public an assessment of eroding democratic regimes in their respective 2020 and 2021 reports analysing the pandemic’s impact on freedom and human rights worldwide (Nazifa et al. 2021). Out of 210 states and territories 112 entities were categorised as partly or not free (Freedom House 2021, p. 17). The Global State of Democracy Report of the International Institute for Democracy and Electoral Assistance (IDEA), issued on 22 November 2021, summarizes the trend in stating that there are now less democracies than autocratic regimes in a global context (IDEA 2021).

In a similar vein, numbers of journalists, publicists but also representatives of epistemic circles in international law and international relations as well as history and military sciences conclude that the withdrawal of the NATO troops from Afghanistan on the 31st of August 2021 signals the end of a Western dominated world and declare illusionary any hope that after the breakdown of the communist regimes a global dominance of liberal democracy would gain place (Goertz 2021, p. 743). The case of Mali seems to underline this conclusion after a consecutive coming into power of military juntas, non-state armed groups like Wagner Group massively operating in the country, and postponed scheduled elections in Spring 2022.

## Afghanistan as a trigger

### Latest facts and figures:

“The deeply disturbing images of the desperate people at Kabul airport” trying to escape the new Taliban regime and its coup d’état-like recapture without any significant resistance of the Afghan government and their security forces are emblematic of the failure of 20 years of Western engagement in Afghanistan (Weiss 2021, p. 8). Media tirelessly frame post-Pashtun Afghanistan as a dark place exiled in a highly volatile and unpredictable future of factional nature (The Guardian 2021). Without any doubt and badly evidenced, the situation resembles a medieval shelter of nursing terrorism and a horror of human rights violations, contrary to what has been the overall goal of all external endeavours of two decades. But things are not as simple as that. The country’s quasi-political lifestyle is coined by diversity and complexity alike, even when the current regime is judged as deteriorating any established degree of freedom and individual self-determination, especially concerning the rights of women and girls (Werther-Pietsch 2021b). Furthermore, what is seen in the West as corruption and clientelism seems to reign as a deep-rooted, given structure of daily business and commercial operations throughout the country.

At the same token, the Afghan economy has a large trade account deficit and is dependent on military spending, foreign aid and access to currency reserves. Aid dependency in Afghanistan, one of the world’s most fragile countries (OECD INCAF 2021; World Bank & United Nations Development Programme 2018), is evident. International financial institutions and the USA unsurprisingly retained massive bank deposits of around US $9 billion when the take-over and the assessment of obvious non-fulfilment of development conditions occurred (Giles et al. 2021). Conditionality is also part of the new strategic response of the European Union, which in the course of the events augmented its support by 200 million € (EU et al. 2021). Simultaneously, in a concerted effort the EU agreed on red lines for cooperation such as the protection of women and girls and female access to primary and higher education, counting for development gains achieved over the last decades (World Bank 2020).

### Decisive speed of social change:

After ending the evacuations and besides continued effort in humanitarian aid, based on local agreements negotiated swiftly after the take-over as well as for refugees in the neighbourhood, the international community is rethinking its short and medium term engagement in and with Afghanistan despite the new regime blatantly countering Western way of life, peace ambitions and values (OECD INCAF 2021)^1^. In fact, long-term development cooperation has been suspended widely, whereas UNDP created a trust fund for Afghanistan in October 2021 to inject liquidity in Afghan households as a first attempt to resume quick recovery for community resilience. This path follows the insight of a meta-evaluation on challenges and dilemmas from Afghanistan (Zürcher et al. 2020) pointing to the fact that smaller, local projects worked best or at least better than non-transparent financial flows, budget support measures, and huge investments in a rapid social change that did not take place.

In regard to the established “Comprehensive Approach” (UNSCR 2016; Werther-Pietsch 2022a, pp. 83–85), this clearly implies that the insight of working together in fragile situations (Austrian Development Cooperation et al. 2011) and respecting the long-term goal of establishing human security has never been implemented on the ground.

When Afghan urban middle class will be unsatisfied in the near future, lacking expected socio-economic development in Afghanistan and pressure on the Taliban regime rises both internally and from the outside, one can imagine that in the end other radical groups could easily be the winners of the new situation (Goertz 2021). Special Representative of the UN Secretary-General for Afghanistan, Deborah Lyons of Canada, told the UN Security Council on November 17, 2021 that the Islamic State-Khorasan Province (ISKP) already appears to be present in all Afghan provinces (Landy 2021). What does this mean in terms of security and development? What can we learn in International Crisis and Conflict Management (ICCM) from Afghanistan? Let us first broaden the picture by an essential facet to complete the prisma.

### Dawn of a new era in geostrategic thinking:

What events in Afghanistan induce is that neighbourhood and the principle of subsidiarity significantly matter to resolute disputes peacefully. Similarly, in the aftermath of the COVID-19 pandemic, an overall appetite of resilience interlinked with vicinity has grown again, culminating in the recent Ukraine war. Is time ripe for a progressive deliberate deconstruction of globalisation and a new peace consensus?

Geostrategic ambitions and delusion have multiplied worldwide since the 9/11 attacks on the World Trade Center in 2001 and, culminating in the veritable contrarius actus now in Afghanistan from May to August 2021, show the emergent need of a new balance-of-power doctrine (Werther-Pietsch 2022a, pp. 173–180, 2022b). To this end, politics of equidistance may be the strategic instrument at hand to advance “principled realpolitik” amid a consolidating bifurcated world (Tchakarova 2021), already embarking on a fierce competition for proxies and spheres of influence: the ultimate USA – China climax. This could not only help disentangling interests of global players involved, such as in the Afghan or, lately, in the Malian (France 24 2022) case, but promote the model of regional strategic autonomy and linked to this, maintain global diversity. The proposed way to find new solutions and make progress is to analyse the potential of future centres of gravity and their interrelations on a global scale. This means exploring, from an EU stance, to what extent a differentiated strategic interface on both universal and regional levels in peace and security can move ahead our common agenda of humanity. The aim is not to step back to territorial concepts of regionalisation but to empower capacity of action through decentralisation.

Furthermore, the sudden end of the international Afghanistan engagement made clear that the EU as a whole, including Germany, is not capable of acting in foreign and security policy without the U.S. (Weiss 2021, p. 8). With the retreat of the USA from its predominant role as the NATO lead nation exercised in Afghanistan the alliance didn’t work any longer. This also comes into play against the background of a clear orientation of the Biden Administration towards the East and the Chinese rivalry in particular. In the words of the EU High Representative Josep Borrell in his foreword to the Strategic Compass, “We need to operate in an increasingly competitive strategic environment” (Borrell 2021; EU & EEAS 2021).

This argument will coin future external engagement, defence planning and capability development of the EU. While NATO may continue to take the lead in territorial defence, the EU and its member states should assume the lead in expeditionary operations where its geostrategic interest is manifest, with NATO in the supporting role, the focus of the EU lying in the Southern flank. Within that framework, the EU could favour an indirect military approach supporting the states through long-term capacity-building, rather than assuming executive tasks itself. Finally, beyond a broad Southern neighbourhood engagement, EU’s military might serve EU diplomacy through port visits, combined exercises, exchange of officers and freedom of navigation operations (Biscop 2021, pp. 21–25). Such conceptualisation also favours an enhanced cooperation between the OSCE and the EU, despite Putin’s war of aggression, as intended in the Strategic Compass of the EU (Frank 2021, p. 674; IIP 2021). This unfolds space for partnerships on a regional basis, particularly in and around Afghanistan.

Responsible sovereignty of regions as a new legal concept comprises a package of mutually reinforcing traits: a renewal of the overall global peace consensus, reinvigorated commitments of non-intervention orchestrated between regions, a refocussed lowest common denominator in universal intervention logic, and primary responsibility of regions when it comes to conflict settlement. Guaranteed regional spheres of autonomous action bound to the joint principle of human centrism may form the basis of a probable future balance-of-power. Trust and honest brokers under the guidance of the UN Secretary-General for such undertaking should be sought for. Since, the dictum of former UN Secretary-General Kofi Annan, “We have come to a fork in the road” (Annan 2003), is valid again.

The thesis of a refocussed intervention logic as the lowest common denominator between a set of regional peace and security drivers is based on the principle of peaceful self-reliance, responsibility and the fight for humanity (Werther-Pietsch 2020, pp. 222–233). Vision-building thereby must be grounded in multilateral system thinking along the milestone-agendas, precising the UN Charter, allowing for and fostering subordinated wings. As stated above, lessons from Afghanistan and maybe Mali shall ideally trigger a renewal of the global peace architecture: The way forward could be framed by more resilient action, localized prioritisation and crisis preparedness in a smaller than global scale or, in other words, to strive for regional strategic autonomy to counteract bipolarism (Werther-Pietsch 2021a, pp. 133–135). This proposal of operational decentralisation of course cannot take place when universal common understanding is lost out of sight.

## Modern forms of ending conflicts by internationalisation

### What is internationalisation and why does it matter?

Issues of international concern today are put on the agenda of the UN Security Council within hours, it only depends on levels of geostrategic interests involved and the respective state of escalation. A potential intervention in latent conflict or fragile situations, such as growing mobilisation in Ethiopia during November 2021 would suggest – be it through fixing of a mandate or through third party mediation – invokes the sudden presence of rules of international law that otherwise wouldn’t have been addressed (Hilpold 2020, p. 98).

Sustained ending of conflicts by internationalisation usually means a peace process conducted politically and/or robustly with the assistance of external actors under close observation by NGOs as watchdogs of humanity (Burgess and Burgess 2010). The famous “domaine reservé” of national sovereignty, however, seems to be shrinking if not closing again (EU et al. 2021) an ever “moving scale” like the Permanent International Court of Justice put it already in 1927. Wolff Heintschel von Heinegg, Wolfgang Nikolaus Graf Vitzthum, Theodor Meron, Kate Parlett, Dieter Fleck, Hans Seidel, Georg Nolte, Johannes Varwick and judges of the International Court of Justice like Sir Gerald Fitzgerald treated this field extensively, elaborating on when a conflict starts/ends, who the acting parties are and what obligations/rights come into play – core issues of what we call international humanitarian law with a focus on jus in bello. But what about the third pillar of law in conflict, the intrinsic jus post bellum, part of which also characterised international engagement in Afghanistan and Mali, which so significantly changed in recent years?

We can certainly discern a clear difference in applicable law as a function of the underlying conceptualisation of sovereignty. The way that has been made from early absolutist perceptions of the principle of sovereign equality (Thomas Aquinas, Hobbes, Locke) via the Age of Enlightenment (Kant, Rousseau, Tocqueville) and the final reinterpretation in the light of responsibility, relativisation of state power and conditional meaning (Deng, Annan, Koskenniemi) shows the dynamic of the world order based on the UN Charter (Paulus 2001). However, there are hard boundaries of international law, one of them being membership, in the presupposed form of “peace loving” statehood alone. It does however not matter which model of legitimacy creates an independent autonomous entity under law (Rocha-Menocal 2011), the decisive fact is the privilege of having seat and voice in the diverse bodies of the UN, solely exercised by states as the primary subjects of international law.

According to the Friendly Relations Declaration (FRD) of 1974 self-proclaimed Afghan leaders of today factually do represent, despite not being freely elected members of government, the Afghan people, and the postulation whether their coming to power was illegal and fulfils substantial elements of an unlawful occupation of a territory can only be retorted by Art. 41. Draft Articles of State Responsibility stipulating that, given the fact of a serious breach of international law, no other state has the right to join in by tolerating it or giving support in any form.^2^ Here we are deeply at the crossroads of pragmatic *realpolitik* and humanitarianism, as we are witnessing in the suburbs of Kiev in early April 2022. This makes the legacy of Afghanistan so preponderant for future developments in international law.

### Categories and intervention models at hand:

Several categories of ending conflict are stipulated in Chapter VI of the UN Charter. According to its Art 33 (1), means of dispute resolution include (Werther-Pietsch 2022a, pp. 46–47):

- Negotiations, good services, mediation
  - Diplomatic means are all forms of direct negotiations between two or more parties under the prerequisite of a fundamental willingness to compromise.
  - “Good offices”, not explicitly mentioned in the Charter, represent diplomatic dispute resolution carried out in the background, such as establishing contacts or providing places for mediating.
  - “Mediation” by third parties (“peace broker”), states, international organisations or private individuals, can also actively lead to positive negotiation results.
- Inquiry, fact finding and reconciliation
  - Settlement of disputes through impartial fact-finding by local or external parties with a non-binding character.
  - Reconciliation proceedings as a kind of mixed form of investigation and mediation, *ad hoc* or contractually.
- Arbitration, recourse to universal or regional judicial regulation
  - Voluntarily accepted arbitration in good faith according to Art. 37 (I) of The Hague Convention for the Peaceful Settlement of International Disputes.
  - Complementary to this, the ICJ as one of the six main organs of the UN can exercise a judicial competence on the basis of its Statute in accordance with Article 7 of the UN Charter, with the right and obligation to interpret the Charter authentically, including acts of the United Nations Supreme Court (UNSC) or regional bodies or agreements. However, the scope of the latter is controversial insofar as the legal significance of resolutions of the UNSC, which are binding for member states, must be taken into account.
  - Other peaceful means such as cultural exchange, sports events, etc. with the aim of contributing to the peaceful settlement of a situation.

To this adds the wisdom of ICCM methods when a post-conflict situation is transformed by peace negotiations and a subsequent peace process, the ultimate challenge for sustainable peace (UNSCR 2016). Although components of resilient peace agreements will vary greatly, some provisions are fairly universal (Smith and Smock 2008, p. 57; see also PA‑X Peace Agreement Databases):

- Constitutionalisation
- Security guarantees
- Demilitarisation, demobilisation, and reintegration
- Protection of all parties’ human rights, especially gender issues
- Return or resettlement of refugees and IDPs
- Social, political, legal, and economic restructuring
- Nature of transitional government
- Elections
- Implementation strategies
- Timetables

This description is by far not exhaustive. The idea here was to offer an overview as a basis to turn to a discussion of a “package of responsibilities” interconnected with the normative emergence of a law of transition in the next chapters.

## “To end a war you need your former enemy” (Gulielmo Verdirame)

### Occupier or liberator:

When issues of power become overwhelming, as Peter Hilpold rightly holds, does the law really has a say? Closely related to just war doctrines (Walzer 2004), this question is a core issue not only in the different phases of hot conflict, it also touches upon the post-conflict scenarios often more challenging, with a view to building up new state structures under a constitutional roof with all the dimensions to be observed on this path. In such endeavours the matter of power nearly automatically regains importance.

The most famous road map to peace among many, UNSCR (1999), for the first one and half a decades after conflict resolution in Kosovo, mandated UNHCR to deal with humanitarian affairs, the UN for the Kosovar Interim Civil Administration, the OSCE for institution-building and the EU for reconstruction. KFOR, the international Kosovo Force, had the tasks of deterring renewed hostilities, establishing a secure environment, ensuring public safety and order, and conducting border monitoring duties (Starlinger 2017, p. 207). This bold experiment, including later steps such as the Ahtisaari Plan 2007 shortly before the declaration of independence of the Kosovar Assembly and the Kosovar President on 17 February 2008, partly reconciled former enemies, with all subsequent stops and go’s we witnessed, 20 years ago in Europe. As Gulielmo Verdirame, following famous peace research tradition (Lederach), stated, wars are no more won on the battlefield but by sustained peacebuilding in the aftermath of conflict (UNSG 2009).

Nevertheless, foreign forces were viewed as occupiers more than as liberators in most cases in history. Too many conditions must be met in case a socio-political life shall be built on the devastated, fuming fields of armed conflict. In these contexts, it seems too narrow an approach when the Stimson doctrine of 1932 or the foundational philosophy of the African Union Charter in 1964 resides in the “uti possidetis” principle. Nor is the sheer prohibition of the use of force, Art. 2 (4) UN Charter, the respective provisions of the 1920 League of Nations as its predecessor or the Briand-Kellog Pact of 1928 sufficient. What is needed today is the unemotional compilation of options for a peaceful together, derived from evidence on how certain circumstances impact the respective normative context, embedded in a few overarching regulations reflecting ius cogens in the realm of universal law (Meron, Stahn, Easterday, Iverson, Bell, Nowak, Thürer).

Concepts of trusteeship or mandates in the past do not seem to have been very successful. Even when, from the end of the (1.) Cold War onwards, the international community went on to act in cases of state failures and governments that lack international recognition or face international sanctions. And this has been proved in the latest Afghan, Libyan or Malian experience. There is still no holistic set of rules for handling transition contexts effectively (Nowak and Werther-Pietsch 2014, pp. 233–234).

### What is needed – holistic strategies in progress:

At least, a few entry points for an “International Law of Transition” are already existent; they will have to be revised and completed along the forthcoming lessons we draw from Afghanistan.^3^ In this context, two new second generation models in peacebuilding and statebuilding inspired by the ICCM concept of “Comprehensive Approach” are outlined below.

*Humanitarian-Development-Peace Nexus:* A very contribution to the above enrolled notion of normativisation of transition is the OECD DAC Recommendation on the Humanitarian-Development-Peace (HDP) Nexus. The decisive step forward in comparison to earlier holistic or integrated approaches is that it is not actor-driven but led by topics. The Nexus Recommendation adopted by the OECD Development Assistance Committee on February 22, 2019 is one of eight comparable instruments of this body. It is not legally binding and represents a political commitment to the principles they contain and entails an expectation that adherents will do their best to implement them.

At the centre of strengthening the coherence between humanitarian, development and peace efforts, is the aim of effectively reducing people’s needs, risks and vulnerabilities, supporting prevention efforts and thus, shifting from delivering humanitarian assistance to ending need. This will be critical in reducing the humanitarian caseload, and ensuring that we meet our collective pledge of “leaving no-one behind”. This requires the engagement of a diverse range of actors, based on their respective comparative advantage, a shared understanding of risk and vulnerability and an approach that prioritises “prevention always, development wherever possible, humanitarian action when necessary” (OECD 2019, p. 3).

Since 2019, the number of parties augmented, with the final result that the UN family is now on board with this experiment, which was lately evaluated by an OECD wide survey, in particular focusing on the critical challenge of integrating the peace pillar into the whole picture.

The Nexus approach, per definition (Art. I), refers to the aim of strengthening collaboration, coherence and complementarity. The approach seeks to capitalize on the comparative advantages of each HDP pillar – to the extent of their relevance in the specific context – in order to reduce overall vulnerability and the number of unmet needs, strengthen risk management capacities and address root causes of conflict. This shall be done in undertaking joint risk-informed, gender-sensitive analysis of root causes and structural drivers of conflict, providing appropriate resourcing to empower leadership for cost-effective coordination across the humanitarian, development and peace architecture, and utilising political engagement and other tools, instruments and approaches at all levels to prevent crises, resolve conflicts and build peace. At the core of the HDP nexus are prevention, mediation and peacebuilding. The Austrian position of 22 October 2021, as a statement on the practical state of implementation – and this links the discussion to Afghanistan and other places – reads as follows:

Given the scarcity of Peace-Pillar activities (4 percent of total DAC ODA), we locate need for more guidance in that context. The draft report is very short on this […], citing roughly conflict prevention, basic safety and security as well as governance and state-building as fields of action, which seems rather superficial. Mediation and peace processes for instance appear to lack totally in this listing. It would be of tremendous value to see more closely which “P”-activities in which situations match best and a deepening of strategic options depending on context if data permits. Related to what Oxfam said on “peace toolboxes”, we therefore encourage further work in that respect.^4^

Localisation as a vision (Santner 2021, pp. 276–279) thereby proves to have been repeated many times as a guiding principle in the past, without however being put into practice and achieved on the ground.

*Mandate of the UN Peacebuilding Commission:* As a remedy of the above-described challenges and failures in transition from “war to peace”, especially in the Afghan case, a re-conceptualisation of the Peacebuilding Commission stemming from 2006 could gain more attention.

With a more and more demanding peace and security agenda, the whole peacebuilding and statebuilding business expanded in the last three decades. In the 1990s, transitional administrations with quasi-state functions developed along operations in Cambodia, East Timor and Kosovo. By means of massive international presence, these engagements *de facto* suspended national sovereignty and the governing power of the state concerned. However, it follows in principle from the logic of collective security as established that once the conflict has been resolved the international mandate ends. From this fact stems the practice of annually extended missions. A further weakness in handling transitions lies in the fact that prolonged post-war contexts as well as protracted crises could only be subsumed under this responsibility in a broad interpretation of intervention to counter fragility (UN High-Level Panel on Threats, Challenges and Change 2004). The Peacebuilding Commission (PBC) finally was founded as a compromise of powers involved in 2005 to institutionally fill this gap between peacekeeping and long-term commitment (UNSCR 2005a, b; UN General Assembly 2005). Originally intended to play a far-reaching role in conflict prevention, the PBC is a subsidiary organ of the two main organs, the UNSC and the UN General Assembly, to which it reports once a year. With a number of fragile states figure on its agenda, the Peacebuilding Fund as the operational instrument has allocated nearly US $1.47 billion to 62 recipient countries from 2006 to 2020.^5^

At the next review, lessons identified from Afghanistan could flow in and be incorporated in a revised bundle of tasks, focusing more on the service of mediating and consulting upon invitation. This would, in accordance with the needs of the people, enable local structures to build their “nation” in a much better harmony with ownership and sustainability.

## The end of nation building through military means

To sum up, it may be useful to reiterate the statement of many development policy experts resorting to the 2005 OECD Paris Declaration that the assumption of a responsibility to rebuild, existing or not (Hilpold 2020, p. 96), should in any case avoid de-responsibilising local actors. Indeed, that is why from a developmental point of view the rebuilders stance always is considered the wrong approach. It might be arguable that an intervening external alliance by fact of its influence and interposition bears a responsibility to also stay engaged when conflict is settled. But, as demonstrated in UNSCR (2011) in the Libyan case, a resolution that suffered false interpretation as giving authorisation for regime change (Feichtinger 2011), early military winnings do not yet guarantee a smooth way to peace. The international community should therefore refrain from “steering” processes of re-instating sovereignty simply because this is a highly sensitive phase where states, societies, local communities and factions fear intervention most, and often fail. As a recent example, the situation in Libya deteriorated steadily, finally ending up in a mess of mercenaries involved. The same could be true for Afghan governance issues of tomorrow.

In 1999, several international organisations like OSCE, EU etc. were involved in the aftermath of an awful war in the Western Balkans. However, and against well-meant premises, a statebuilding process, carried out with strong foreign assistance, runs the risk of being qualified as the result of a foreign intervention and therefore be denied recognition by people concerned (Hilpold 2020, p. 97). What has been falsified in Libya, Kosovo, Afghanistan and elsewhere, is that military cannot substitute political transition, broad space must be given to all other players in the civil sphere, good and bad guys, as they are: “Ever tried, ever failed. No matter. Try again, fail again, fail better”, as Samuel Beckett argued. For this reason, regarding modern perceptions of peace processes, Thania Paffenholz worked out the notion of “perpetual peacebuilding” operating through dialogue and mindful advising (Videos UWP 2021).

## Preliminary conclusions

### Learning from the German example:

At the peak of German engagement in Afghanistan (2006–2010), around 150,000 German armed forces were present in the country (Gauster, 2021, p. 12), compared to 85,000 NGO representatives (Moballegh 2021). 1300 German soldiers were deployed in the last period of the NATO operations Resolute Support (2015–2021) aiming at staffing and equipping the Afghan army to stabilise their own country. During the mission, which “defended Germany at the hights of the Hindukush” (Jahn 2015, pp. 209–224), 59 troops lost their lives, and more of them were left injured and traumatised. The total costs of German military engagement amounted up to 12 billion €. The initial success of “Operation Enduring Freedom” in destroying safe harbours of al-Qaida, similar the killing of the organisation’s leader Bin Laden in Pakistan in 2011, could have been taken as a victory. This is particularly bound to, according to the mandates of UNSCR (2001) on spontaneous self-defence and Art. 5 NATO, declaring an attack on one of its members an attack, on all. However, the mission later became enlarged by the International Security Assistance Force (ISAF). ISAF with staff from 51 nations and from 2003 onwards, supported by the much-criticised Provincial Reconstruction Teams (PRTs), figures as an early model of structured civil-military cooperation. However, the overhasty withdrawal of all NATO allies upon the U.S. leave, as mentioned in Chapter 2.3, showed limitations of partnership with especially logistical and other warfare capabilities lying only with the U.S.

Networked security, a policy concept embracing the insight of deliberately combining diplomatic, economic, police and military in fragile situations in order to save any chance of success, must not exist on paper alone. So, the incident of two empty flights of the Gesellschaft für Internationale Zusammenarbeit (GIZ) back from Kabul under siege this year revealed that Germany obviously did not dispose of a common list for evacuation of local staff because responsible ministries took different approaches. Consequentially, a national security council as a coordination mechanism, as suggested by Stefani Weiss from the Bertelsmann Stiftung, could probably remedy the situation and press for more coherent action (Weiss 2021, p. 9; Werther-Pietsch 2020, 2022b).

### Are the whole-of’s totally passé?

This question remains difficult terrain, especially with a view to Mali or Eastern-Ukraine. From the very beginning the double-hated approach, to secure and rebuild on the one hand, and countering insurgency and terrorism (COIN) on the other, became a crucial test for the international alliance in Afghanistan. Where the U.S.’s point of view of merging the two objectives was basically rejected by EU members and other partners, they inversely felt forced to agree as allies. Tellingly, the UN Assistance Mission in Afghanistan, a civilian mission of the UN, very much in line with the Europeans, did never see a comparable equipment or resources. This recently changed when projects in the framework of the Chinese Belt and Road Initiative met with Afghan recovery programmes (Gauster 2021, p. 13).

The agreement of Doha of 29 February 2020, under former U.S. President Trump and with participation of the German Special Representative for Afghanistan, marked a historical turn in the U.S. engagement. His successor, President Biden followed this path of confining terrorist acts: no “exporting of” and no more harbouring should be undertaken by the Taliban, no threat to U.S. soil be exercised. However, this seemingly was made under the silent presumption that the Taliban regime would not be able to sweep away the Western-backed Pashtun government in Kabul, a presumption that was overwhelmingly falsified sur place end of August 2021. To conclude, and assessing steady change of objectives of NATO’s successive Afghanistan mission, it definitely turned out that the predominance of military targets overruled civilian goals, so that in the end “no one seemed to know exactly why NATO was involved in Afghanistan: Was it the war on terror? Or […] primarily a civil reconstruction mission that served to develop a democratic state oriented towards Western values?” (Weiss 2021, p. 8).

### Is, then, any engagement illusionary?

Let us try to delineate future recommendations by distinction of cases and intervention levels. In doing so, Johannes Varwick (2021, pp. 318–319) aptly contributes four decisive points to scrutinize past failures:

- Concert of actors: Lacking strategic and jointly developed preparedness including road maps with options, differing features, exit plans, and flexible scenarios;
- Timeline: short, medium and long-term thinking should be interlinked as demonstrated in the HDP nexus, including collateral or unintended effects;
- A change of mindset from the “occupier” to the “enabler”: responsibility to rebuild as a task for locals is what is asked for. This is linked to the question whether any attempt of democratisation and statebuilding from the outside is feasible from the bottom up without reflecting the three factors of ownership, the primacy of policy and modesty (OECD 2011), and – perhaps a German specificity;
- Avoidance of the so-called “antibiotic intervention”: acknowledgement of the humanitarian imperative and simultaneous under-resourcing respectively understaffing of missions, so that they, stuck half-way, cannot succeed from the very beginning.

A fifth point may be added with a view to Ukraine. In the face of the breakdown of a long-standing international engagement in Afghanistan, it seems appropriate to take further Peter Hilpold’s considerations to a full-fledged agreed package of “shared responsibilities” of the international community, the regional level and the country concerned, each of them normatively based on the same raison d’être, peace:

- “Responsibility to prevent”;
- “Responsibility to mediate”;
- “Responsibility to protect”, existing/emerging since 2005; and
- “Responsibility to rebuild”.

The goal of European branded democratic state building was born in the residential quarter of Petersberg in Bonn, mainly initiated and conducted by Germany, as early as 2001. Later, this way distinguished by its compatibility and inspiration from development policy conceptions, was reinforced by the OECD Dialogue on Peacebuilding and Statebuilding in 2008–2011 (IDPS 2022). Given the length of mostly military presence in Afghanistan and the many obvious setbacks due to electoral fraud, corruption, clientelism and a secure situation that had never been stabilised (Weiss 2021, p. 8), this path should have been brought to life much earlier. There is no doubt that, from the Libyan scenario and beyond, fundamental questions of external behaviour in crisis and conflict zones will have to be rethought in any expeditionary operation such as, in the near future, in Mali with France withdrawing from soil. Or, hopefully, one day in Ukraine when peace will be reinstalled.

Interestingly, at the end of his argumentation Johannes Varwick appears to find a consolidated fair balance in the final perception of the Afghan exercise, a view weighing chances that might have materialised through the international engagement, the minimum of human rights upheld, successful restrictions to export violence beyond borders, development gains. He thereby refers to an underlying complex finding: whenever realists and idealists face each other in the international arena, it is the extent to which power can be limited and fenced by the principle of human centrism and peaceful change that counts (UNSG 2020).

## Footnotes

1 Further information on this source is available through the author upon request.

2 “Art. 41. Particular consequences of a serious breach of an obligation under this Chapter: 1. States shall cooperate to bring to an end through lawful means any serious breach within the meaning of Art. 40. 2. No State shall recognize as lawful a situation created by a serious breach within the meaning of Art. 40, nor render aid or assistance in maintaining that situation. 3. This article is without prejudice to the other consequences referred to in this Part and to such further consequences that a breach to which this Chapter applies may entail under international law.” (UN 2001).

3 The OECD International Network on Conflict and Fragility has mandated a Task Team on 18 November 2021 to conduct a comprehensive evaluation of the Afghanistan missions, to be published in September 2022.

4 Further information on this source is available through the author upon request.

5 The data is available at United Nations (2022).
